# Supplementary material for: From sequence to enzyme mechanism using multi-label machine learning
Source: BMC Bioinformatics. 2014 May 19;15:150. doi: 10.1186/1471-2105-15-150 (PMC4229970; doi:10.1186/1471-2105-15-150)
Supplement: Additional file 2 — Java code of ml2db. Additional file ml2db_code.tar.gz contains the Java source code to run the multi-label machine learning experiments and save the results to database. The code’s Javadoc is included. [file 1471-2105-15-150-S2.zip › additional file 2/ml2db/ml2db/doc/main/ml2db/al/utility/pickers/class-use/Picker.html]

Uses of Interface main.ml2db.al.utility.pickers.Picker


JavaScript is disabled on your browser.


- Overview
- Package
- Class
- Use
- Tree
- Deprecated
- Index
- Help

- Prev
- Next

- Frames
- No Frames

- All Classes

## Uses of Interface main.ml2db.al.utility.pickers.Picker

- Packages that use Picker

  | Package | Description |
  | --- | --- |
  |  |  |
  | --- | --- |
  | main.ml2db.al.utility.pickers |  |
  | main.ml2db.al.utility.pickers.byfrequency |  |
- - ### Uses of Picker in main.ml2db.al.utility.pickers

    Classes in main.ml2db.al.utility.pickers that implement Picker

    | Modifier and Type | Class and Description |
    | --- | --- |
    |  |  |
    | --- | --- |
    | `class` | `LabelRandomPicker` Class |
    | `class` | `RandomAttributePicker` Picks an attribute uniformly randomly and then one of the unlabelled instances having that attribute. |
    | `class` | `RandomAttributeSetPicker` Class |
    | `class` | `RandomInstancePicker` Class |
    | `class` | `RandomLabelsPicker` Class |
    | `class` | `RandomNeigbourhoodPicker` Class |
    | `class` | `RandomPicker` Class |
  - ### Uses of Picker in main.ml2db.al.utility.pickers.byfrequency

    Classes in main.ml2db.al.utility.pickers.byfrequency that implement Picker

    | Modifier and Type | Class and Description |
    | --- | --- |
    |  |  |
    | --- | --- |
    | `class` | `AttributesByFrequencyPicker` Class |
    | `class` | `AttSetByFrequencyPicker` Serves the instances in order of frequency of their attribute sets (most frequent first). |
    | `class` | `ByFrequencyPicker` Picks each of the features (class, attribute or attribute set) by descending order of frequency, and then, uniformly randomly, one of the instances of that feature. |
    | `class` | `LabelsByFreqPicker` Class |
    | `class` | `NearestNeighboursByFreqUtility` Class |

- Overview
- Package
- Class
- Use
- Tree
- Deprecated
- Index
- Help

- Prev
- Next

- Frames
- No Frames

- All Classes
